# Molecular analysis of type 3 fimbrial genes from *Escherichia coli*, *Klebsiella *and *Citrobacter *species

**DOI:** 10.1186/1471-2180-10-183

**Published:** 2010-06-24

**Authors:** Cheryl-lynn Y Ong, Scott A Beatson, Makrina Totsika, Christiane Forestier, Alastair G McEwan, Mark A Schembri

**Affiliations:** 1 Centre for Infectious Disease Research, School of Chemistry and Molecular Biosciences, University of Queensland, Brisbane, Queensland 4072, Australia; 2 Université de Clermont 1, UFR Pharmacie, Laboratoire de Bactériologie, 28 place Henri Dunant, Clermont-Ferrand, F-63001 France

## Abstract

**Background:**

Catheter-associated urinary tract infection (CAUTI) is the most common nosocomial infection in the United States and is caused by a range of uropathogens. Biofilm formation by uropathogens that cause CAUTI is often mediated by cell surface structures such as fimbriae. In this study, we characterised the genes encoding type 3 fimbriae from CAUTI strains of *Escherichia coli*, *Klebsiella pneumoniae*, *Klebsiella oxytoca*, *Citrobacter koseri *and *Citrobacter freundii*.

**Results:**

Phylogenetic analysis of the type 3 fimbrial genes (*mrkABCD*) from 39 strains revealed they clustered into five distinct clades (A-E) ranging from one to twenty-three members. The majority of sequences grouped in clade A, which was represented by the *mrk *gene cluster from the genome sequenced *K. pneumoniae *MGH78578. The *E. coli *and *K. pneumoniae mrkABCD *gene sequences clustered together in two distinct clades, supporting previous evidence for the occurrence of inter-genera lateral gene transfer. All of the strains examined caused type 3 fimbriae mediated agglutination of tannic acid treated human erythrocytes despite sequence variation in the *mrkD*-encoding adhesin gene. Type 3 fimbriae deletion mutants were constructed in 13 representative strains and were used to demonstrate a direct role for type 3 fimbriae in biofilm formation.

**Conclusions:**

The expression of functional type 3 fimbriae is common to many Gram-negative pathogens that cause CAUTI and is strongly associated with biofilm growth. Our data provides additional evidence for the spread of type 3 fimbrial genes by lateral gene transfer. Further work is now required to substantiate the clade structure reported here by examining more strains as well as other bacterial genera that make type 3 fimbriae and cause CAUTI.

## Background

Catheter-associated urinary tract infection (CAUTI) is the most common nosocomial infection in the United States and a frequent cause of bacteremia [1]. Nosocomial CAUTI is caused by a range of different bacterial pathogens [2] and these are often resistant to multiple antibiotics [3].

Biofilm formation is a trait commonly found among CAUTI isolates and results in the growth of bacteria on the inner surface of the urinary catheter. Biofilm formation promotes encrustation and protects the bacteria from the hydrodynamic forces of urine flow, host defenses and antibiotics [4]. A perquisite to biofilm growth is adherence to the catheter surface. A number of mechanisms by which Gram-negative pathogens mediate adherence to biotic and abiotic surfaces have been described and include fimbriae (e.g. type 1, type 3, type IV, curli and conjugative pili), cell surface adhesins (e.g. autotransporter proteins such as antigen 43, UpaH and UpaG) and flagella [5-16].

The expression of type 3 fimbriae has been described from many Gram-negative pathogens [17-28]. Type 3 fimbriae are 2-4 nm wide and 0.5-2 μm long surface organelles that are characterised by their ability to mediate agglutination of tannic acid-treated human RBC (MR/K agglutination) [29]. Several studies have clearly demonstrated a role for type 3 fimbriae in biofilm formation [17,28,30-33]. Type 3 fimbriae also mediate various adherence functions such as binding to epithelial cells (from the respiratory and urinary tracts) and extracellular matrix proteins (e.g. collagen V) [31,34-36].

Type 3 fimbriae belong to the chaperone-usher class of fimbriae and are encoded by five genes (*mrkABCDF*) arranged in the same transcriptional orientation [29,37]. The *mrk *gene cluster is similar to other fimbrial operons of the chaperone-usher class in that it contains genes encoding major (*mrkA*) and minor (*mrkF*) subunit proteins as well as chaperone- (*mrkB*), usher- (*mrkC*) and adhesin- (*mrkD*) encoding genes [37,38]. A putative regulatory gene (*mrkE*) located upstream of *mrkA *has been described previously in *Klebsiella pneumoniae *[37]. The *mrk *genes have been shown to reside at multiple genomic locations, including the chromosome [39], on conjugative plasmids [17,30] and within a composite transposon [40]. Transfer of an *mrk*-containing conjugative plasmid to strains of *Salmonella enterica *serovar Typhimurium, *Klebsiella pneumoniae*, *Enterobacter aerogenes *and *Kluyvera *species has also been demonstrated [17]. Taken together, these data strongly support spread of the *mrk *genes between Gram-negative pathogens by lateral gene transfer.

Recently, we identified and characterised the role of type 3 fimbriae in biofilm formation from an *Escherichia coli *strain isolated from a patient with CAUTI [28]. We also demonstrated that the *mrkB *chaperone-encoding gene and the ability to mediate MR/K agglutination was common in uropathogenic *Klebsiella pneumoniae*, *Klebsiella oxytoca *and *Citrobacter koseri *strains (86.7%, 100% and 100% of strains, respectively) but rare in uropathogenic *E. coli *and *Citrobacter freundii *strains (3.2% and 14.3% of strains, respectively) [28]. Despite the occurrence of type 3 fimbriae genes among a range of different Gram-negative bacteria that cause CAUTI, little is known about their molecular relationship. In this study, we have examined the phylogenetic correlation between type 3 fimbrial (*mrk*) genes from 33 CAUTI strains representing five different uropathogens (*E. coli*, *K. pneumoniae*, *K. oxytoca*, *C. koseri *and *C. freundii*). We also demonstrate functional expression of type 3 fimbriae in each of these strains and describe a common role for type 3 fimbriae in biofilm formation.

## Results

### Phylogenetic analysis of the *mrkABCD *genes from uropathogenic bacterial genera

**Figure 1:**
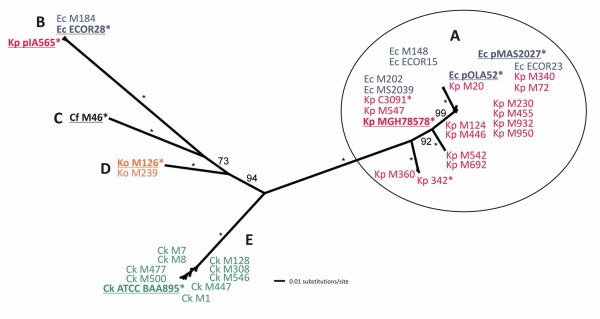
**Unrooted consensus phylogram of the concatenated *mrkABCD *nucleotide fragments**. Majority-rule consensus tree was based on 500 bootstrap replicates using dnaml, the DNA maximum likelihood algorithm implemented by PHYLIP [54]. Five well-supported clades are labelled A-E; the largest clade, A, is circled. Bootstrap values are shown; small asterisks next to branches denote 100% support. Taxon IDs include species name abbreviations as suffixes (Cf, *C. freundii *indicated in black; Ck, *C. koseri *indicated in green; Ec, *E. coli *indicated in blue; Ko, *K. oxytoca *indicated in orange; and Kp, *K. pneumoniae *indicated in red), followed by the strain name. Taxon IDs highlighted in bold and underlined refer to those used in further analyses of the complete sequence of their respective *mrk *locus. Complete *mrk *locus sequences available from GenBank are marked with a large asterisk next to the strain name.

To investigate the phylogenetic relationship of the *mrk *genes from 33 CAUTI strains (representing *E. coli, K. pneumoniae, K. oxytoca, C. koseri *and *C. freundii*) we amplified and sequenced an internal segment of the *mrkA*, *mrkB*, *mrkC *and *mrkD *genes from each strain. We also examined the corresponding sequence from six additional *mrk *gene clusters available at GenBank. A majority-rule consensus maximum likelihood (ML) tree was constructed from the 39 concatenated *mrkABCD *fragments. The phylogenetic analysis indicated that the sequences clustered into five major clades (referred to as clade A to E) with good bootstrap support (Fig. 1). The five clades range from one member (clade C, represented by *C. freundii *M46) to 23 members (clade A, represented by *K. pneumoniae *MGH78578), with an average inter-allelic diversity of 11.2%. Whereas the 10 *C. koseri *sequences clustered in a single clade (clade E), clade B (3 sequences) and clade A (23 sequences) consist of sequences from both *K. pneumoniae *and *E. coli*. Phylogenetic analysis using parsimony or distance-based methods produced tree topologies very similar to those obtained by using DNA maximum likelihood (data not shown).

The incongruence between the *mrk *consensus tree and the established phylogeny for enteric bacteria [41] is *prima facie *evidence for lateral gene transfer (LGT) of *mrk *alleles. All *K. pneumoniae *chromosomal alleles cluster in Clade A, along with several plasmid-borne or chromosomal alleles from *E. coli*. In some cases, the *K. pneumoniae *and *E. coli *alleles are identical (e.g. Ec_pOLA52/Kp_M20; EcM202/Kp_MGH78578). Similarly, in clade B, two identical *E. coli mrkABCD *sequences (M184 and ECOR28) share high nucleotide sequence identity (98%) to the plasmid-borne *K. pneumoniae *pIA565 *mrkABCD*. The *mrkABCD *concatenated nucleotide sequences of *K. pneumoniae *pIA565 (clade B) and *K. pneumoniae *MGH78578 (clade A) share only 78.2% nucleotide sequence identity. When analysed individually, the *mrkA, mrkB, mrkC *and *mrkD *gene fragment alignments produced essentially the same tree topology as the concatenated sequence (data not shown) with little variation in within-group diversity (Table 1). In contrast to other chaperone-usher systems, the *mrkD *adhesin is more divergent than the *mrkA *major subunit and contributes the most of all *mrk *alleles to the inter-group diversity (Table 1).

**Table 1:** Diversity of individual *mrkA, mrkB, mrkC *and *mrkD *nucleotide sequences

		**Diversity Within Group (%)**^**1**^			**Diversity Between Group (%)**^**2**^			
Gene	Length	A	B	E	Mean	A and B	A and E	B and E
*mrkA*	403 nt	2.3	0.2	2.5	13.8	14.7	15.6	11.8
*mrkB*	246 nt	1.0	0.8	1.3	9.8	12.2	14.0	8.9
*mrkC*	655 nt	2.1	0.3	0.6	13.5	18.4	19.6	12.2
*mrkD*	506 nt	3.3	0.3	0.3	28.1	38.2	26.7	33.3

^1^Mean within group diversity; Group C and Group D excluded as they contain a single sequence, and two identical sequences, respectively.

^2^Mean between group diversity calculated with all five groups.

### Sequence comparison of the *mrk *locus from strains of *C. freundii*, *C. koseri*, *E. coli *and *K. oxytoca*

**Figure 2:**
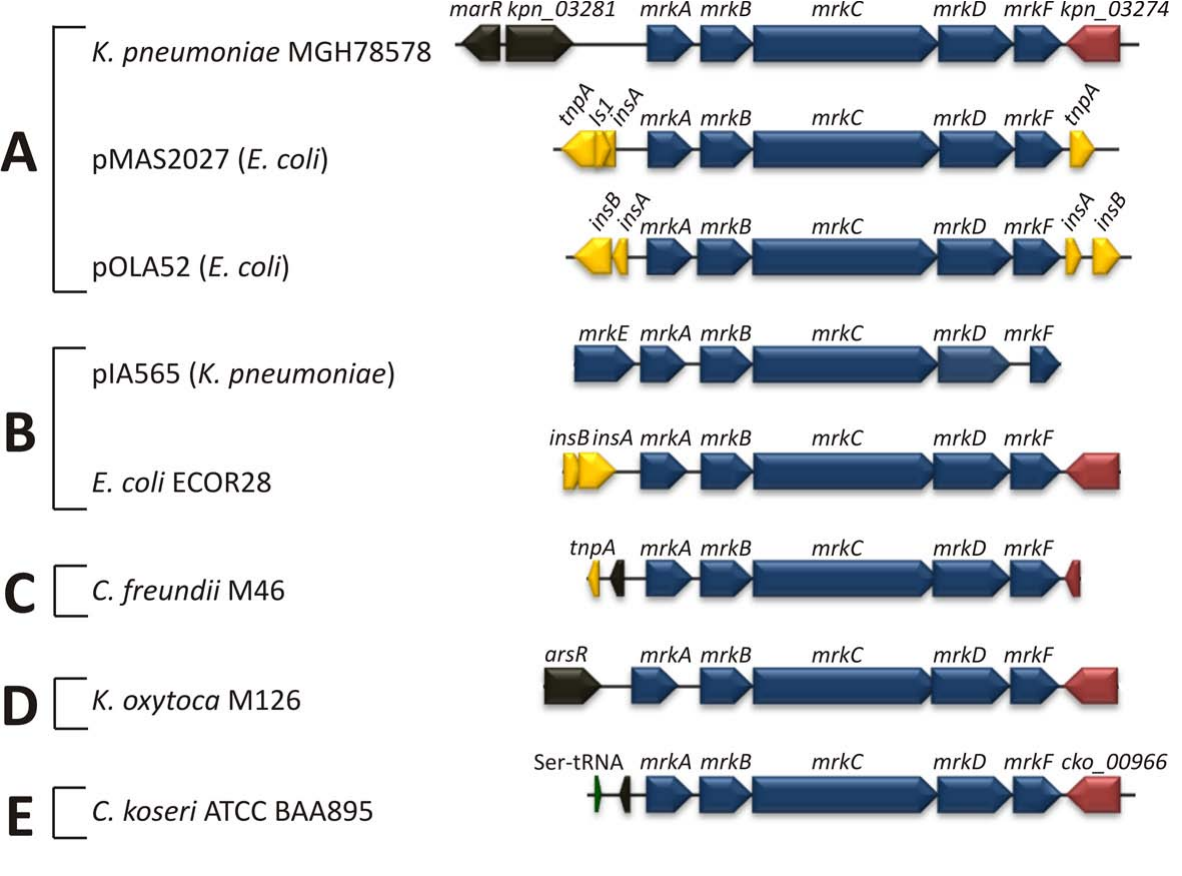
**Genetic organisation of the type 3 fimbriae (*mrk*) gene cluster**. Physical map of the *mrk *gene cluster and flanking regions from *K. pneumoniae *MGH78578, plasmid pMAS2027 (*E. coli*), plasmid pOLA52 (*E. coli*), plasmid pIA565 (*K. pneumoniae*), *E. coli *ECOR28, *C. freundii *M46, *K. oxytoca *M126, and *C. koseri *ATCC BAA895. The *mrk *genes are indicated in blue and include *mrkE *(putative regulatory gene), *mrkA *(major subunit encoding gene), *mrkB *(chaperone encoding gene), *mrkC *(usher encoding gene), *mrkD *(adhesin encoding gene) and *mrkF *(putative minor subunit encoding gene). ORFs encoding putative transposable elements (yellow) and hypothetical proteins (grey) are indicated. The gene indicated in red (labelled cko_00966 and kpn_03274 in the genomes of *C. koseri *ATCC BAA895 and *K. pneumoniae *MGH78578, respectively) encodes a hypothetical protein containing a central EAL domain and was present downstream of *mrkF *in 29 strains. Sequence information outside the *mrk *cluster is not known for *K. pneumoniae *pIA565. Arrows indicate the direction of transcription for each gene.

We compared the *mrk *gene clusters from representatives of each of the five clades: 5 *mrk *regions were available from GenBank, 3 were sequenced in this study (Fig. 2). As expected, the *mrkABCD *gene order is conserved in all clades. Predicted insertion sequences were identified flanking both ends of the pMAS2027 and pOLA52 clusters (clade A), and at the 5' end of clusters from ECOR28 (clade B) and *C. freundii *M46 (clade C), indicative of recent lateral gene transfer. Downstream of *mrkF*, a conserved 717 bp gene was present in five of the strains, including one from each of the five defined clades. This gene (labelled cko_00966 and kpn_03274 in the genomes of *C. koseri *ATCC BAA895 and *K. pneumoniae *MGH78578, respectively) encodes a central EAL domain (Pfam:PF00563, E = 1.7e-29) suggesting that it may have a role in signalling, however, no close homologs have been functionally characterised. PCR primers designed from these sequences demonstrated that this region was also conserved in 24 other strains examined (data not shown). Notably, cko_00966 homologs were not encoded downstream of the plasmid-borne *mrk *clusters in *E. coli *pMAS2027 and pOLA52, and there is no corresponding sequence information available for this region in pIA565 [37]. The putative *mrkE *regulatory gene originally identified in pIA565 [37] was not present in any of the strains examined.

### Type 3 fimbriae are functionally expressed in *C. freundii*, *C. koseri*, *E. coli*, *K. oxytoca *and *K. pneumoniae*

**Figure 3:**
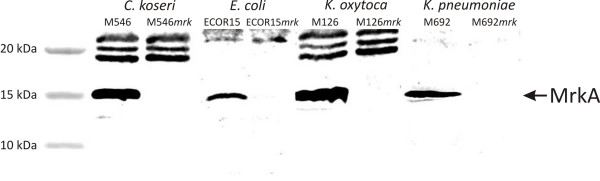
**Western blot analysis employing type 3 fimbriae specific antiserum**. The proteins from *C. koseri *M546 (lane 2), *C. koseri *M546*mrk *(lane 3), *E. coli *ECOR15 (lane 4), *E. coli *ECOR15*mrk *(lane 5), *K. oxytoca *M126 (lane 6), *K. oxytoca *M126*mrk *(lane 7), *K. pneumoniae *M692 (lane 8) and *K. pneumoniae *M692*mrk *(lane 9) were acid boiled prior to loading. Molecular size markers are indicated in lane 1. The Type 3 fimbriae major subunit, MrkA, was only observed in the wild-type strains and not in the *mrk *deletion mutants. The arrow indicates the ~15 kDa band corresponding to MrkA.

**Figure 4:**
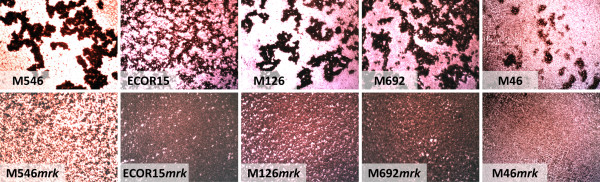
**Phase contrast microscopy illustrating MR/K agglutination**. Parental wild-type strains *C. koseri *M546, *E. coli *ECOR15, *K. oxytoca *M126, *K. pneumoniae *M692 and *C. freundii *M46 demonstrated strong agglutination of tannic acid treated human erythrocytes, while their corresponding *mrk *deletion mutants, M546*mrk*, ECOR15*mrk*, M126*mrk*, M692*mrk *and M46*mrk *were negative for agglutination.

**Figure 5:**
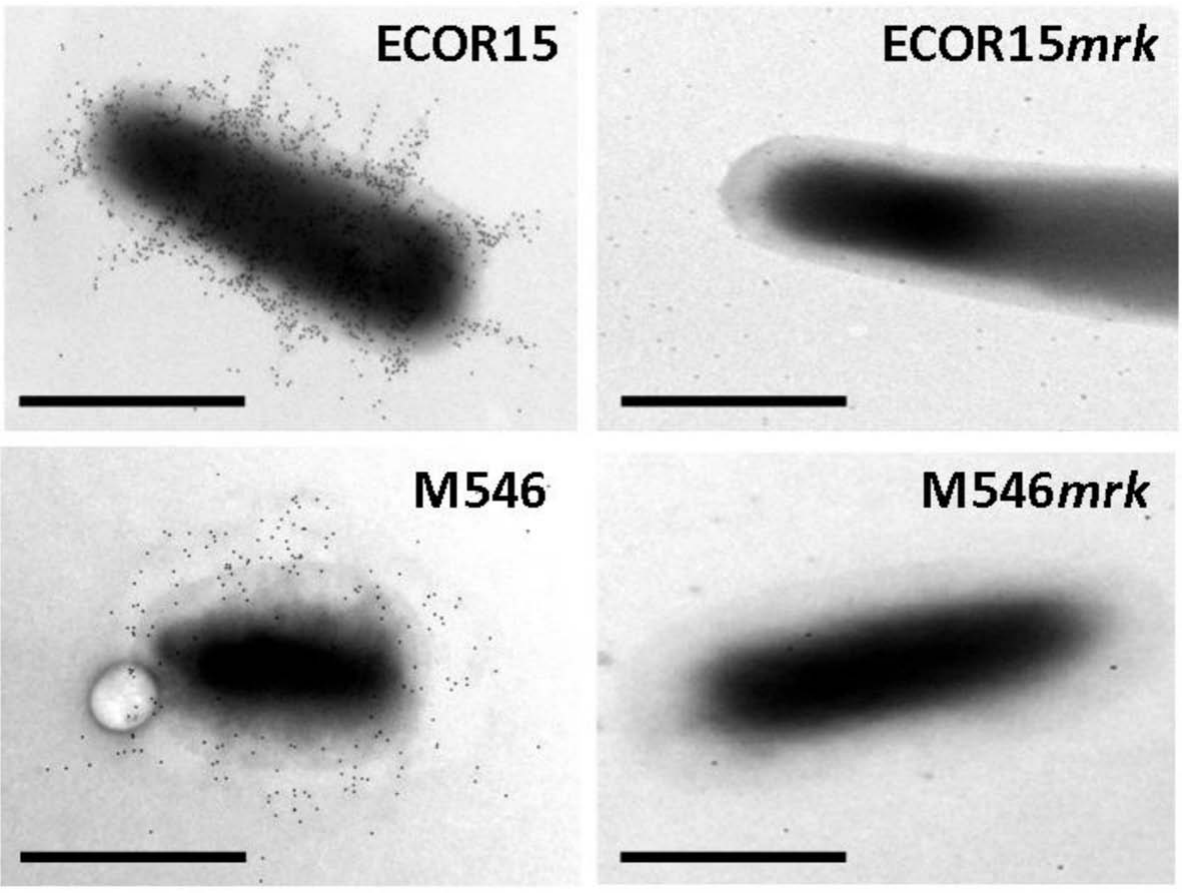
**Immunogold electron microscopy demonstrating expression of type 3 fimbriae in *E. coli *ECOR15 and *C. koseri *M546**. Expression of type 3 fimbriae at the cell surface was demonstrated by abundant labelling with anti-type 3 fimbriae-gold particles. In contrast, the deletion mutants, *E. coli *ECOR15*mrk *and *C. koseri *M546*mrk *were virtually devoid of gold labelling. Scale bar represents 1 μm.

All of the *mrk*-positive strains examined in this study mediated mannose-resistant hemagglutination of tannic acid treated human RBC (MR/K agglutination), indicating they produced type 3 fimbriae. To specifically demonstrate a direct association between MR/K agglutination and type 3 fimbriae, the *mrk *locus was deleted from thirteen strains (*E. coli *MS2027, M184, ECOR15, ECOR28; *K. pneumoniae *M20, M124, M446, M542, M692; *K. oxytoca *M126, M239; *C. freundii *M46; *C. koseri *M546) employing *λ*-red mediated homologous recombination. The strains were selected on the basis of their transformation efficiency and included at least one representative from each of the *mrk *phylogenetic clades. Several different assays were employed to compare the thirteen sets of wild-type and *mrk *deletion strains. First, SDS-PAGE analysis of crude cell lysates and subsequent Western blotting was performed using a type 3 fimbriae-specific antiserum. A predominant 15 kDa band representing the MrkA major subunit was detected from all wild-type strains except *C. freundii *M46, which failed to react positively in this assay. In contrast, no reaction was observed for any of the *mrk *deletion mutants (Fig. 3 and data not shown). Next, the wild-type and *mrk *mutant strains were compared for their ability to mediate MR/K agglutination. Only the wild-type strains produced a positive phenotype (Fig. 4 and data not shown). Finally, the presence of type 3 fimbriae was confirmed by immunogold labelling employing type 3 fimbriae-specific antiserum for *E. coli *ECOR15 and *C. koseri *M546, but was absent in their corresponding *mrk *deletion mutants (Fig. 5). Taken together, the results demonstrate that MR/K agglutination is a conserved phenotype for a range of Gram-negative organisms that express functional type 3 fimbriae.

### Type 3 fimbriae are strongly associated with biofilm formation

**Figure 6:**
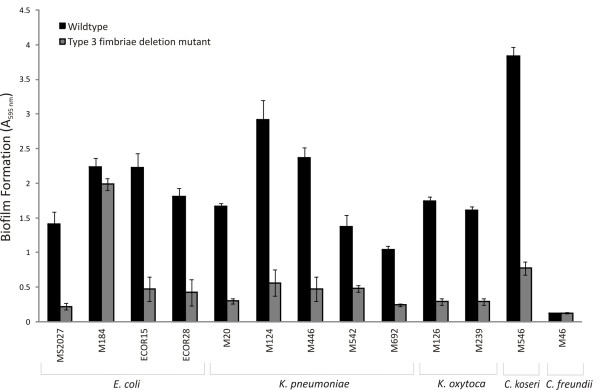
**Biofilm formation by wild-type and isogenic *mrk *deletion strains**. Strains were grown at 37°C under shaking conditions for 16 h in PVC microtitre plates containing M9 minimal medium, washed to remove unbound cells and stained with 0.1% crystal violet. Biofilm formation was quantified by resuspending adherent cells in ethanol-acetate (80:20) and measuring the absorbance at 595 nm. Shown are the results for *E. coli *MS2027, M184, ECOR15 and, ECOR28, *K. pneumoniae *M20, M124, M446, M542 and, M692, *K. oxytoca *M126 and, M239, *C. koseri *M546 and *C. freundii *M46 and their respective *mrk *deletion mutants.

The thirteen sets of isogenic wild-type and *mrk *deletion strains generated above were examined for their ability to produce a biofilm following growth in M9 minimal medium (containing 0.2% glucose) under dynamic culture conditions. Strong biofilm growth was observed from all wild-type strains except *C. freundii *M46. In contrast, deletion of the *mrk *gene cluster caused a significant reduction in biofilm growth (*p *< 0.0001) in all strains except *E. coli *M184 (Fig. 6). Similar results were also observed following growth in synthetic urine (data not shown). Thus, type 3 fimbriae contribute significantly to biofilm formation when expressed in *E. coli, K. pneumoniae, K. oxytoca *and *C. koseri*.

## Discussion

Type 3 fimbriae are adhesive organelles produced by a range of Gram-negative pathogens that cause CAUTI. Here we show that type 3 fimbriae (*mrkABCD*) genes from 33 CAUTI isolates representing *C. freundii*, *C. koseri*, *E. coli*, *K. oxytoca *and *K. pneumoniae *cluster into five well-supported clades on the basis of nucleotide sequence. Type 3 fimbriae were expressed by all of these strains as indicated by their positive MR/K agglutination. Type 3 fimbrial expression was also associated with biofilm growth in the majority of these strains. This is the first report describing the distinct grouping of type 3 fimbrial genes into phylogenetic clades at the species level, with strong evidence supporting inter-species lateral gene transfer. We also demonstrate the functional expression of type 3 fimbriae by strains of *C. koseri *and *C. freundii*.

Phylogenetic analysis with individual and concatenated *mrkABCD *sequences revealed five distinct clades (A-E) which were strongly supported by long internal branches. The majority of the sequences grouped in clade A, which is represented by the chromosomal *mrk *gene cluster from the genome sequenced *K. pneumoniae *strain MGH78578. Clades A and B contained *mrk *gene clusters from *K. pneumoniae *(both chromosomal and plasmid origin) and *E. coli *(plasmid origin). Two *mrk *loci have been fully sequenced from *E. coli*; in both cases the *mrk *genes are located on a conjugative plasmid (pMAS2027 and pOLA52, respectively) and flanked by transposon-like sequences [30,40]. While the genomic location of the *mrk *genes in the additional seven *E. coli *strains identified in this study remains to be determined, the data presented here and in previous studies strongly suggests inter-genera lateral gene transfer of the *mrk *cluster [17,28]. In contrast, the composition of clade E is entirely *C. koseri *sequences, while clades C and D are represented by a unique sequence from *C freundii *and *K. oxytoca*, respectively. The presence of cko_00966 homologs downstream of representative *mrk *clusters in all 5 clades strongly suggests that the ancestral *mrkABCD *locus was also encoded next to a cko_00966 homolog and that the clades are largely related by linear descent. Notably, the relationship determined here is not congruent with the known evolutionary relationship of *Klebsiella*, *Citrobacter*, and *E. coli *[41], supporting the occurrence of lateral gene transfer. We propose that clade A represents the *K. pneumoniae *lineage, with *mrk *regions laterally transferred to *E. coli *(e.g. pMAS2027 and pOLA52) and clade E represents the *C. koseri *lineage. Clades B, C and D, which contain *mrk *sequences from *K. pneumoniae, E. coli, C. freundii *and *K. oxytoca*, are clearly under-represented and additional type 3 fimbrial gene sequences are required to confirm the groupings.

Among the four genes used in the phylogenetic analysis, *mrkD *exhibited the highest inter-group diversity (Table 1). Thus, from the partial sequence comparisons performed in this work, the MrkD adhesin displayed greater sequence variability than the MrkA major subunit. This is inconsistent with other chaperone-usher fimbriae such as type 1 and P fimbriae, where the sequence of the adhesin (e.g. FimH, PapG) is more conserved than the major subunit protein (e.g. FimA, PapA). We note, however, that these findings require substantiation via comparison of the entire sequence of each structural subunit from multiple strains. The MrkD adhesin mediates several phenotypes, including MR/K agglutination, as well as adherence to human endothelial cells, urinary bladder cells, basement membranes and ECM proteins such as collagen IV and V [5,31,34,35]. Interestingly, previous studies have demonstrated that sequence variations in the MrkD adhesin are associated with differential binding properties [42-44]. Our study demonstrates that the degree of sequence variation in MrkD might be even greater than previously predicted [44].

CAUTI is associated with biofilm formation on the inner surface of indwelling catheters. Thirteen independent *mrk *deletion mutants were generated and used to examine type 3 fimbriae associated phenotypes including MR/K agglutination and biofilm formation. All of the *mrk *mutants were unable to cause MR/K agglutination, confirming that this property is highly specific for type 3 fimbriae. In biofilm assays, 11/13 *mrk *mutants displayed a significant reduction in biofilm growth compared to their respective parent strain, demonstrating that type 3 fimbriae contribute to this phenotype across a range of different genera and species. The exceptions were *C. freundii *M46 and *E. coli *M184. *C. freundii *M46 failed to produce a significant biofilm in the assay conditions employed irrespective of its *mrk *genotype. Although this strain caused MR/K agglutination, we were also unable to detect the MrkA major subunit protein by western blot analysis. *E. coli *M184 showed no reduction in biofilm growth upon deletion of the *mrk *genes. It is likely that *E. coli *M184 contains additional mechanisms that promote biofilm growth and therefore deletion of the *mrk *genes did not result in loss of this phenotype.

## Conclusions

This study demonstrated that the expression of functional type 3 fimbriae is common to many Gram-negative pathogens that cause CAUTI. Biofilm growth mediated by type 3 fimbriae may be important for the survival of these organisms on the surface of urinary catheters and within the hospital environment. Although our analysis provides additional evidence for the spread of type 3 fimbrial genes by lateral gene transfer, further work is required to substantiate the clade structure reported here by examining more strains as well as other genera that make type 3 fimbriae and cause CAUTI such as *Proteus *and *Providentia*.

## Methods

### Bacterial strains, plasmids & growth conditions

The strains and plasmids used in this study are described in Table 2. Clinical UTI isolates were obtained from urine samples of patients at the Princess Alexandra Hospital (Brisbane, Australia) and have been described previously [45]. *E. coli *ECOR15, ECOR23 and ECOR28 were from the *E. coli *reference (ECOR) collection [46]. Cells were routinely grown at 37 °C on solid or in liquid Luria-Bertani (LB) medium supplemented with appropriate antibiotics unless otherwise stated. M9 minimal medium and synthetic urine were formulated as previously described [47,48].

**Table 2:** Bacterial strains and plasmids used in this study

*Strains/Plasmids*	*Description*	*Reference*
		
**Strains**		
MS2027	*E. coli *CAUTI isolate	[28]
M20	*K. pneumoniae *CAUTI isolate	[28]
M46	*C. freundii *ABU isolate	[28]
M124	*K. pneumoniae *CAUTI isolate	[28]
M126	*K. oxytoca *CAUTI isolate	[28]
M184	*E. coli *pyelonephritis isolate	[28]
M239	*K. oxytoca *CAUTI isolate	[28]
M446	*K. pneumoniae *CAUTI isolate	[28]
M542	*K. pneumoniae *CAUTI isolate	[28]
M546	*C. koseri *CAUTI isolate	[28]
M692	*K. pneumoniae *CAUTI isolate	[28]
MS2181	CAUTI *E. coli *MS2027*mrk::cam*	This study
MS2266	Pyelonephritis *E. coli *M184*mrk*::*cam*	This study
MS2267	*E. coli *ECOR15*mrk*::*cam*	This study
MS2332	CAUTI *K. pneumoniae *M124*mrk*::*kan*	This study
MS2334	CAUTI *K. pneumoniae *M446*mrk*::*kan*	This study
MS2335	CAUTI *K. pneumoniae *M542*mrk*::*kan*	This study
MS2374	CAUTI *K. pneumoniae *M20*Δrk*::*kan*	This study
MS2377	CAUTI *K. oxytoca *M126*mrk*::*kan *	This study
MS2379	CAUTI *K. oxytoca *M239*mrk*:: *kan *	This study
MS2454	CAUTI *C. koseri *M546*mrk*::*kan*	This study
MS2456	ABU *C. freundii *M46*mrk*::*kan*	This study
MS2458	*E. coli *ECOR28*mrk*::*kan*	This study
MS2515	CAUTI *K. pneumoniae *M692*mrk*::*kan *	This study
		
**Plasmids**		
pKD3	Deletion mutant template plasmid (*cam*)	[49]
pKD4	Deletion mutant template plasmid (*kan*)	[49]
pKD46	Temperature-sensitive plasmid containing λ-Red recombinase system	[49]
pKOBEG199	Plasmid with λ-Red genes under the control of the arabinose-inducible promoter	[50]

### DNA manipulations and genetic techniques

Plasmid DNA was isolated using the QIAprep Spin Miniprep Kit (Qiagen, Australia). Restriction endonucleases were used according to the manufacturer's specifications (New England Biolabs, USA). Chromosomal DNA was purified as previously described [48]. PCR was performed using *Taq *polymerase according to the manufacturer's instructions (New England Biolabs, USA). DNA sequencing was performed by the Australian Equine Genome Research Centre. Deletion mutants were constructed essentially as previously described using either pKD46 [49] or pKOBEG199 [50,51], with the exception that *C. freundii *and *C. koseri *strains were heated at 42°C for 2 min prior to electroporation. Primers used to generate deletion mutants were as follows: 1293 and 1294 (*E. coli *MS2027), 1456 and 1457 (*E. coli *ECOR15 and *K. pneumoniae *strains), 1458 and 1459 (*E. coli *ECOR28), 1456 and 1459 (*E. coli *M184), 1460 and 1459 (*K. oxytoca *strains), 1456 and 1461 (*C. koseri *M546), 1462 and 1459 (*C. freundii *M46) (Table 3). All deletion mutants were checked by PCR using specific primers (Table 3) in conjunction with primers targeting the kanamycin or chloramphenicol resistance gene [49] and further confirmed by sequencing. Sequence information outside the *mrk *cluster was obtained by inverse PCR (using primer combinations 1450/1452, 1450/1454, 1450/1453, 1451/1455, or 1451/1453) or standard PCR employing primers designed from the genome sequenced *K. pneumoniae *MGH78578 or *C. koseri *ATCC BAA895 (Table 3).

**Table 3:** Primers used in this study

Primer	Description	Sequence (5'-3')
**Type 3 fimbriae deletion primers**		
1293	50 bp overhang *mrk *knockout F-primer 1	TCTTCTCTCTGCAGCAATGGCAACCGCGTTTTTTGGCATGACTGCTGCCCGTGTAGGCTGGAGCTGCTTCG
1294	50 bp overhang *mrk *knockout R-primer 1	GGTGTGAGCGGGATAGTTGTCTGAGTCACAGGCAGTTTCCTCTTCACCAGCATATGAATATCCTCCTTAG
1295	Knockout screening F 1	GGCAGCATAACCGAACAAAT
1296	Knockout screening R 1	TAAATTTTCTGCGGCAAACC
1456	50 bp overhang *mrk *knockout F-primer 2	GTTAACGGTACCCGCTTTATTTATCCAGGAAATGAAAAAGAAATAACGGTGTGTAGGCTGGAGCTGCTTCG
1457	50 bp overhang *mrk *knockout R-primer 2	CCTTTGTCCCAGAACTCCGGGCTGACATAGTTTTTCAGGCGTTGATCTTCCATATGAATATCCTCCTTAG
1463	Knockout screening R 2	GGTCTGGTTGCTGTTCCAGT
1458	50 bp overhang *mrk *knockout F-primer 3	GAGAGCTGCACCGTTATTTCTTTTTCATTTCCTGGATAAATAAAGCGGGTGTGTAGGCTGGAGCTGCTTCG
1459	50 bp overhang *mrk *knockout R-primer 3	CTTTACCGGCGGTGATTTTCAGGTCGTAGCGCCCTTTCCATTCGCCGTTGCATATGAATATCCTCCTTAG
1464	Knockout screening F 3	CTGCCGAGCTTAATACGCA
1465	Knockout screening R 3	GCAACGCCTTTATCCCAGA
1460	50 bp overhang *mrk *knockout F-primer 4	CTGCCGTTCAGGACGGCGCGCGCTTCACTTACTACGTTGGCTACGCTACCGTGTAGGCTGGAGCTGCTTCG
1461	50 bp overhang *mrk *knockout R-primer 4	GTGTAGCAGACGCCCATTTTGCCGTCTTTTCCACGGGTGATATTCAGGTCCATATGAATATCCTCCTTAG
1466	Knockout screening F 4	ATCCAGCTGGTTCTGTCCAC
1467	Knockout screening R 4	CCGGGCTGACAAAATTCTTA
1462	50 bp overhang *mrk *knockout F-primer 5	GGCTGGATAACGGTAATGCTGACGCAACGCCGGATACTATTACGACTCCAGTGTAGGCTGGAGCTGCTTCG
1468	Knockout screening F 5	AAAAGAAATAACGGTGCAGCTC
**Inverse PCR primers**		
1450	mrkA R-primer A	CTTTACCGAAGAAATTAACC
1451	mrkA R-primer C	GAAGAAATTAACCTGACCGCC
1452	mrkD F-primer A	AAACCTATCTGAGCGCCAAC
1453	mrkD F-primer B	CCTCTTATGACTGGGAGAACG
1454	mrkD F-primer C	TGCGCGCTTCTATCAATATG
1455	mrkD F-primer D	GGCGTCCAGGTACTGAAAGA
**Outside *mrk *cluster screening primers**		
1469	F-primer (outside *mrkA *of *K. pneumoniae *MGH78578)	TGGCGGTACATACTTCACTCA
1470	R-primer (outside *mrkF *of *K. pneumoniae *MGH78578)	ATCCTCTGCCTATTGTCTGACT
1471	F-primer (*mrkE *of *K. pneumoniae *pIA565)	TTTGCATATCCGCAATCTGA
1472	F-primer (outside *mrkA *of *C. koseri *ATCC BAA-895)	AATTGCAGGAACAGGGTCTG
1473	R-primer (outside *mrkF *of *C. koseri *ATCC BAA-895)	TTCCTTTCCCGTAACCA
1474	F-primer (outside *mrkA *of *E. coli *MS2027)	AGCGTTCTGCATGGCTTTAT
1475	R-primer (outside *mrkF *of *E. coli *MS2027)	AACGACGTTGGGTCGTTAGT
**pMAS2027 screening primers**		
1476	pMAS2027 screening primer F1	GCGCAGAACTGGTTATGAAT
1477	pMAS2027 screening primer R1	TCATGGATTTTCTTCCTTAACAA
1478	pMAS2027 screening primer F2	ACAACTATCCCGCTCACACC
1479	pMAS2027 screening primer R2	ACCGTTAACGCGTAGTCACC
1480	pMAS2027 screening primer F3	TGCTTCAGCAGCATATCAGG
1481	pMAS2027 screening primer R3	GGAAAGCGTTAAAGCAGGTG
1482	pMAS2027 screening primer F4	CCGTATGCGCTTTTTCAAGT
1483	pMAS2027 screening primer R4	AAAGTTGAAGCCCGCTTTCT
1484	pMAS2027 screening primer F5	ACGGGTAAGACCGCTAACCT
1485	pMAS2027 screening primer R5	TCGATAAGGTAGGCATCAACAA
		

### MR/K agglutination

Bacterial agglutination of tannic acid treated human erythrocytes (MR/K agglutination) was performed as previously described to detect the expression of Type 3 fimbriae [29]. Bacterial strains were grown overnight as shaking cultures in M9 minimal medium. Strains which produced a negative result in this assay were enriched for type 3 fimbriae production by three successive rounds of 48 h static growth in M9 minimal medium and then re-tested.

### Biofilm study

Biofilm formation on polyvinyl chloride (PVC) surfaces was monitored by using 96-well microtitre plates (Falcon) essentially as previously described [16]. Briefly, cells were grown for 24 h in M9 minimal medium (containing 0.2% glucose) or 48 h in synthetic urine at 37°C under shaking conditions, washed to remove unbound cells and stained with crystal violet. Quantification of biofilm mass was performed by addition of acetone-ethanol (20:80) and measurement of the dissolved crystal violet at an optical density of 595 nm. All experiments were performed in a minimum of eight replicates.

### Immunoblotting and immunogold-labelled electron microscopy

Crude cell lysates were prepared from overnight cultures and boiled in acid as previously described [14]. Protein samples were analysed by SDS-PAGE and western blotting as previously described [52] employing a type 3 fimbriae specific antiserum. Immunogold labelling was performed using the same Type 3 fimbriae specific antiserum as previously described [14]. Cells were examined under a JEOL JEM1010 TEM operated at 80 kV. Images were captured using an analysis Megaview digital camera.

### Phylogenetic and sequence analysis

PCR products were generated from an internal region of *mrkA *(416 bp), *mrkB *(243 bp), *mrkC *(657 bp) and *mrkD *(778), respectively, from each of the 33 CAUTI strains and sequenced on both strands. These sequences correspond to nucleotides 112 to 530 of *mrkA*, 66 to 308 of *mrkB*, 173 to 829 of *mrkC *and 157 to 934 of *mrkD *in the reference strain *K. pneumoniae *MGH78578 (CP000647). Individual and concatenated gene fragments from the 33 CAUTI strains (and six additional *mrk *sequences available at GenBank from strains causing other infections; accession numbers: CP000647, EU682505, CP000964, M55912, CP000822, EU370913) were aligned using ClustalX [53], and subjected to phylogenetic analysis using PHYLIP [54]. Maximum likelihood (ML) trees were built from a concatenated alignment of 2104 nucleotides (comprising 1269 conserved sites and 775 informative sites) using the dnaml algorithm in PHYLIP [54]. A consensus tree of 500 ML bootstrap replicates was prepared using the majority rule method as implemented by Splitstree version 4 [55,56]. We were unable to amplify *mrkD *from *E. coli *M202 and only used the *mrkABC *concatenated fragments in the analysis. For comparative analysis, the complete *mrk *cluster (and adjacent regions) from *E. coli *ECOR28, *C. freundii *M46 and *K. oxytoca *M126 were amplified using an inverse PCR strategy and sequenced.

### Statistical analysis

Differences in biofilm formation between wild-type and *mrk *mutant strains were analysed using the ANOVA single factor test (Minitab 15 Statistical Software).

### Nucleotide sequence accession numbers

Gene fragments were deposited in GenBank under the accession numbers: FJ96754, FJ96756-FJ96774, and FJ96777-FJ96789 (for *mrkA*), FJ96793, FJ96795-FJ96811, FJ96813-FJ96814, and FJ96817-FJ96829 (for *mrkC*) and FJ96832, FJ96834-FJ96849, FJ96851-FJ96852, and FJ96855-FJ96867 (for *mrkD*). The *mrkB *sequences were described previously [28]. The complete *mrk *cluster (and adjacent regions) from *E. coli *ECOR28, *C. freundii *M46 and *K. oxytoca *M126 were deposited in GenBank under accession numbers FJ96870, FJ96871 and FJ96872, respectively.

### Ethical approval

Approval for this study was obtained from the Princess Alexandra Hospital Human Research Ethics Committee (2005/098). Since the study used *E. coli *isolates collected as part of routine methods for the diagnosis of UTI and no additional procedures on patients were involved, individual informed consent was not obtained.

## Authors' contributions

CYO carried out the majority of the experimental work under the supervision of AGM and MAS. SAB performed the bioinformatic analysis and contributed to writing the manuscript. MT performed the immunogold labelled electron microscopy and contributed to writing the manuscript. CF contributed to the construction of mutants and writing of the manuscript. AGM contributed to the design of experiments and writing of the manuscript. MAS conceived the study and wrote the manuscript. All authors read and approved the final manuscript.
